# Viroid Intercellular Trafficking: RNA Motifs, Cellular Factors and Broad Impacts

**DOI:** 10.3390/v1020210

**Published:** 2009-09-01

**Authors:** Ryuta Takeda, Biao Ding

**Affiliations:** Department of Plant Cellular and Molecular Biology and Molecular, Cellular and Developmental Biology Program, Ohio State University, 207 Rightmire Hall, 1060 Carmack Road, Columbus, 43210 USA; E-Mail: takeda.7@buckeyemail.osu.edu

**Keywords:** viroid, RNA motif, RNA trafficking, plasmodesmata, phloem

## Abstract

Viroids are noncoding RNAs that infect plants. In order to establish systemic infection, these RNAs must traffic from an initially infected host cell into neighboring cells and ultimately throughout a whole plant. Recent studies have identified structural motifs in a viroid that are required for trafficking, enabling further studies on the mechanisms of their function. Some cellular proteins interact with viroids *in vivo* and may play a role in viroid trafficking, which can now be directly tested by using a virus-induced gene silencing system that functions efficiently in plant species from which these factors were identified. This review discusses these recent advances, unanswered questions and the use of viroid infection as an highly productive model to elucidate mechanisms of RNA trafficking that is of broad biological significance.

## 1. Introduction

Viroids are single-stranded, circular, noncoding and infectious RNAs in plants that have genome sizes ranging from 246 to 401 nucleotides [1–5]. Currently known viroids are classified into two families: Avsunviroidae and Pospiviroidae, based on their sequences, structures and biological properties [2]. Briefly, the members of Avsunviroidae, with type species being *Avocado sunblotch viroid* (ASBVd), have a quasi-rod-like secondary structure with branching, possess intrinsic ribozyme activities and replicate in the chloroplast. The members of Pospiviroidae, typified by *Potato spindle tuber viroid* (PSTVd), have a rod-like secondary structure, lack evident ribozyme activities and replicate in the nucleus. After replication within a cell, some viroid progeny move into neighboring cells to initiate new rounds of replication so as to spread infection, provided that a host is compatible for systemic infection. Depending on viroid-host combinations, an infected plant may be symptomless or develop severe disease symptoms including plant death.

In this special issue, Flores and colleagues provide an updated account of the replication mechanisms for the two families of viroids [6]; Owens and Hammond [7] discuss recent progress on the mechanisms of viroid pathogenicity; and Elena and colleagues [8] address intriguing evolutionary questions of viroids. Here we focus on the intercellular trafficking of viroids, reviewing current knowledge of RNA motifs and potential cellular factors in trafficking. We highlight unresolved questions regarding regulation of viroid trafficking and their potential for broadening our understanding of pathogen-host interaction mechanisms. We conclude by illustrating the exceptional quality of viroid trafficking as a model to help uncover the molecular machinery that traffic cellular and infectious RNAs.

## 2. The pathway of viroid intercellular trafficking

**Figure 1.**
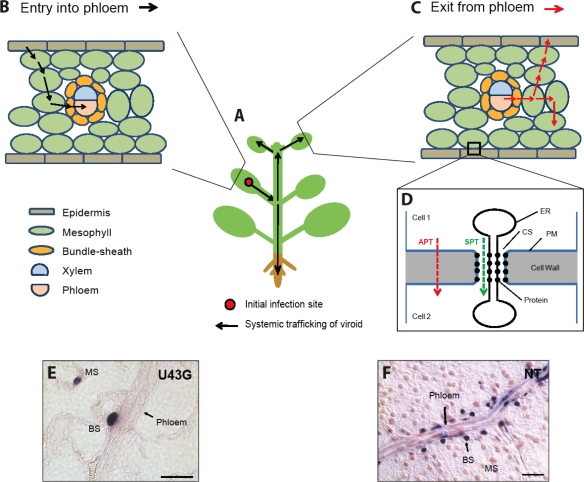
Pathways for systemic trafficking of a viroid in an infected plant and genetic analyses of viroid RNA motifs required for trafficking along these pathways. **A.** A simplistic drawing of a plant in which PSTVd infection initiated locally in an inoculated leaf can spread to the upper, systemic leaves as well as the root. **B.** A simplistic drawing of a leaf showing cell-to-cell trafficking of a viroid from an initially infected epidermal cell to the phloem to embark on long-distance transport into other organs to spread infection. For simplicity, different cell types in the mesophyll, xylem and phloem are not illustrated. **C.** In a systemic leaf, the viroid will exit the phloem and traffic into other nonvascular cells. **D.** A diagram showing cell walls that separate two cells with a plasmodesma allowing direct cell-to-cell symplasmic transport (SPT). The plasma membrane permits exchange of some molecules across the cell walls via apoplastic transport (APT). **E–F.** Genetic identification of viroid motifs required for entering the phloem from bundle sheath in an inoculated leaf and exiting the bundle sheath to invade mesophyll in a systemically infected leaf, respectively (See Section 3 for details). The images show longitudinal sectional views of the phloem. The purple dots represent hybridization signals of PSTVd RNA in the nuclei. **E.** In situ localization of mutant U43G in mesophyll (MS), bundle sheath (BS) and absence from the phloem in an inoculated leaf of *N. benthamiana*. **F.** In situ hybridization shows presence of PSTVd^NB^ in phloem, bundle sheath (BS) and mesophyll (MS) and PSTVd^NT^ in only the phloem and bundle sheath (BS), respectively, in a systemically infected tobacco leaf. (Image E is adapted from [20] with permission from American Society of Plant Biologists. Image F is adapted from [21] with permission from Nature Publishing Group). CS, cytoplasmic sleeve; ER, endoplasmic reticulum. Scale bars=10μm.

Plant cells have walls that form a physical barrier for free movement of molecules between cells. Intercellular molecular exchange can occur via two alternative pathways: plasma membrane-mediated apoplastic transport and plasmodesma-mediated symplasmic transport. Plasmodesmata are plasma membrane-bound cytoplasmic channels that offer open and gatable pores between neighboring cells for direct cell-to-cell transport (Figure 1). A cylinder of modified endoplasmic reticulum (ER) runs through the middle of each pore, creating the so-called cytoplasmic sleeve between the ER and plasma membrane that form micro-channels for the passage of molecules. Within the plasma membrane and ER are embedded proteins that may play structural and regulatory roles [9]. Long-distance symplasmic transport of molecules occurs via the vascular tissue phloem, specifically the sieve tubes in the phloem that comprise enucleate living cells at maturity and are interconnected end to end to form long transport channels [10].

Viroid intercellular trafficking does not occur via plasma membrane-mediated transport, but rather occurs via plasmodesmata. This was demonstrated by monitoring the cell-to-cell spread of fluorescent-labeled *in vitro* transcripts of PSTVd injected into mesophyll cells that are interconnected by plasmodesmata or into mature guard cells that have no plasmodesmal connections with neighboring cells in a leaf. In the former case, the viroid transcripts rapidly spread from cell to cell; whereas in the latter case the viroid transcripts never left the injected cell [11]. For long-distance spread between different plant organs, a viroid utilizes the phloem, as shown by the similar movement patterns in a whole plant between PSTVd and photoassimilates [12] and by direct in situ localization of PSTVd to the phloem [13].

## 3. Genetic identification of viroid motifs for intercellular trafficking

In order to identify viroid structural motifs critical for intercellular trafficking, it is important to define the technical criteria first. A most direct and compelling criterion is genetic mutation in a viroid RNA structural element that abolishes or inhibits trafficking in a plant but does not abolish replication in single cells, thereby identifying the structural element affected by the mutation as a motif critical for trafficking. The merit of this criterion is the confident identification of motifs important for trafficking versus those important for replication. This is particularly important for a viroid RNA in which all functions are expressed from the RNA itself. A caveat of this criterion is that some motifs may play a role in both trafficking and replication, thereby making it difficult or impossible to separate these functions. Furthermore, some types of mutations introduced into certain genomic positions may cause structural modifications that affect the overall function of the RNA to cloud interpretations of results. Despite these potential complications, recent studies have successfully identified a range of structural motifs in the PSTVd genome that play a critical role in trafficking. Furthermore, detailed studies of some motifs indicate their requirement for trafficking between specific cell types. These advances, discussed below, start to shed light on the viroid structural elements critical for trafficking and build a basis for further discoveries in this direction.

### 3.1. A genome-wide mutational analysis identified a range of PSTVd trafficking motifs

**Figure 2.**
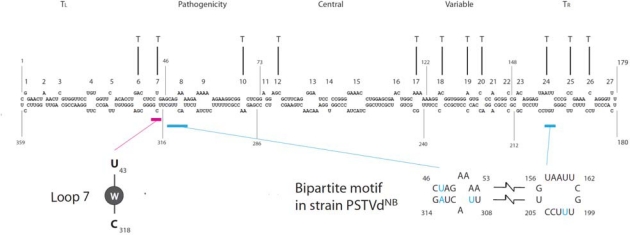
Secondary structure of PSTVd and genomic locations of trafficking motifs (T). Loop 7, which comprises a U/C *cis*-WC/WC base pair with water insertion and which is required for bundle sheath-to-phloem trafficking, is based on Zhong *et al*. [20]. The bipartite motif for bundle sheath-to-mesophyll trafficking is based on Qi *et al*. [21]. All other trafficking motifs are based on Zhong *et al*. [18]. The five structural domains of PSTVd are based on Keese and Symons [51]. T_L_, left-terminal domain; T_R_, right-terminal domain.

As shown in Figure 2, the secondary structure of PSTVd consists of a series of loops/bulges (collectively called loops thereafter for convenience of description) flanked by short helices. Helices are formed by canonical *cis*-Watson-Crick (WC)/WC basepairing. Based on X-ray crystallographic and nuclear magnetic resonance studies showing that most loops in an RNA secondary structure often form three-dimensional (3D) motifs via non-WC basepairing, base stacking and other base-base interactions [14] and that such motifs often function in RNA-RNA, RNA–protein and RNA-small ligand interactions [14–17], it is reasonable to postulate that the various loops in PSTVd and other viroids are functional motifs.

Using loss-of-function genetic approaches, Zhong and colleagues identified various loops in PSTVd required for systemic trafficking in experimental host *Nicotiana benthamiana* (*N. benthamiana*) [18]. Each loop was obliterated by nucleotide deletions or closed by nucleotide deletions/substitutions that result in the formation of canonical *cis*-WC/WC base pairs without altering the overall rod-like secondary structure as predicted by Mfold [19]. Each mutant was then tested for its capacity to infect a plant systemically as well as to replicate in single cells (i.e., protoplasts which are prepared by enzymatic removal of cell walls). Accumulation of a mutant in a systemic leaf above the inoculated leaf, with the original mutations in the viroid progeny confirmed by sequencing, indicates its competency for systemic trafficking. Lack of accumulation of a mutant in a systemic leaf, with its replication competency confirmed in protoplasts, indicates defects in trafficking function. Such analyses identified a total of 10 mutants, each carrying one obliterated loop, defective in systemic infection (Figure 2, indicated as T; see discussion on loop 7 below). These are classified as “trafficking-defective” mutants, and the loops affected by the mutations are therefore designated as motifs essential for trafficking. Another 9 mutants, affecting other loops, failed to infect systemically 80–90% of the inoculated plants. In a small percentage of the plants that are systemically infected, the viroid progeny exhibit additional mutations and/or reversion to the wild type sequences. Therefore, they are classified as “trafficking-impaired” mutants. The biological importance, specifically their impact on trafficking, of the sequence reversions and additional mutations in these mutants remains to be determined.

This work shows that most loops in PSTVd play a role in trafficking. The current collection of trafficking mutants provides a useful resource for experimentation to identify the cellular boundary at which each trafficking motif functions, the 3D structure of a motif and the cellular factor(s) that interacts with a motif trafficking. It will be of great interest to determine whether other viroids also possess multiple motifs to mediate their intercellular trafficking.

### 3.2. Loop 7 has a defined tertiary structure and is required for phloem entry

Detailed genetic, structural and cellular studies determined that loop 7 (U43/C318, Figure 2) is required for PSTVd trafficking from bundle sheath into the phloem in inoculated *N. benthamiana* leaves [20] (Figure 2). Nucleotide substitutions U43G and C318A, respectively, that close the loop by creating canonical *cis*-WC/WC basepairing abolished PSTVd systemic infection, but not replication in protoplasts or inoculated leaves. In situ hybridization localized the two mutants to epidermal, mesophyll and bundle sheath cells, but not in any vascular cells of an inoculated leaf, indicating the requirement for loop 7 to enable PSTVd to enter the phloem (see Figure 1E for mutant 43G). RNA structural database searches with a 3D motif search program found recurring U/C motifs in crystal structures of rRNAs and other RNAs, which is characterized by *cis*-WC/WC basepairing with water insertion. Water insertion distorts the local helix, creating a pocket for protein binding. The PSTVd U43/C318 motif is postulated to be similarly structured (Figure 2), which is supported by results from analyzing the trafficking capacity of all possible 15 additional mutants. All mutants that maintain trafficking function have high local water density within the base pair and all mutants with low water density lose trafficking function. This motif most likely serves a protein-binding site in PSTVd. Identifying the protein involved should yield additional insights into the mechanisms of trafficking.

A similar motif exists in other viroids in the genus Pospiviroid, suggesting its similar function in these viroids [20]. It is expected that similar genetic, structural and cellular approaches will enable detailed studies on the functions of other trafficking motifs identified from the genome-wide mutational analysis [18]. Such analyses should help address the question of whether U43/C318 motif functions alone or together with additional motifs for bundle sheath-to-phloem trafficking. In a broader sense, such analyses should be extended to other viroids in order to develop an understanding of how viroids have evolved mechanisms to utilize host systems to establish systemic infection [1].

### 3.3. A bipartite motif is required for trafficking from bundle sheath to mesophyll

An important question is whether a single or multiple motifs is required for trafficking of a viroid across a specific cellular boundary. There is already an example of complex motifs for unidirectional trafficking across a cellular boundary [21]. Two PSTVd strains, PSTVd^NT^ and PSTVd^NB^, differ by five nucleotides. One difference is in the right-terminal region and the other four in the pathogenicity region. PSTVd^NB^ accumulates more than PSTVd^NT^ in systemically infected tobacco leaves, although they show similar replication levels in protoplasts. In situ hybridization showed presence of PSTVd^NB^ in all cell types, but restriction of PSTVd^NT^ to the phloem and bundle sheath cells, of a systemic leaf (see Figure 1F for PSTVd^NT^). This indicates the inability of the latter to traffic from bundle sheath into mesophyll and beyond. Nucleotide swapping between the two strains showed that four of the five nucleotide differences are responsible for the competency of PSTVd ^NB^ to traffic out of bundle sheath cells [21]. The four nucleotides (U201, U309, U47/A313) in PSTVd^NB^ strain therefore constitute a so-called bipartite motif that is required for trafficking from the bundle sheath to mesophyll, but not required for trafficking in the opposite direction (Figure 2).

Several outstanding questions remain to be answered: Do the two parts of this motif interact intra-molecularly or function independently? Do the two parts each interact with a different cellular protein(s)? Is it a specialized motif in PSTVd^NB^ for tobacco infection or does it have some general implications?

## 4. Cellular factors in viroid trafficking

Because viroid intercellular trafficking is mediated by specific structural motifs rather than by diffusion, a simple and generic mechanistic model would predict interactions between these motifs with cellular factors. Thus far, however, no cellular factors that function in viroid trafficking have been conclusively identified, for instance through loss-of-function means. Genetic identification of such factors is not technically feasible at the present time, because all known viroid hosts are not amenable to productive mutagenesis screening. Under such circumstances, biochemical identification of viroid-interacting proteins followed by functional studies by targeted gene knockout or knockdown will remain a major approach.

Biochemical method has allowed identification of a number of mobile phloem proteins that interact with some viroids. These include the cucumber phloem lectin CsPP2 and melon phloem lectin CmmLec17. CsPP2 interacts with *Hop stunt viroid* (HSVd) both *in vitro* and *in vivo* [22–24] and CmmLec17 binds ASBVd [25]. A 14 kDa protein of unknown biochemical nature from the melon phloem also interacts with ASBVd [25]. To provide conclusive evidence for the requirement of these proteins in viroid trafficking, it should be demonstrated that knockout or knockdown expression of each of them negatively impacts viroid trafficking. Recently, a series of *Apple latent spherical virus* vectors have been developed that enable RNA interference (RNAi)-mediated efficient gene silencing in cucumber and other cucurbits [26]. This system may provide a powerful means to inhibit expression of viroid-interacting proteins in these species to directly test the role of a cellular protein in viroid trafficking.

In a recent exciting development, Ham *et al*. [27] isolated a phloem-mobile ribonucleoprotein complex from pumpkin for the selective trafficking of some cellular RNAs. It will be interesting to test whether this complex, or some component(s) of this complex, plays a role in viroid trafficking.

## 5. Future prospects

As discussed, most progress on viroid trafficking motifs has been made with analyses of PSTVd. Some PSTVd trafficking motifs recur in some other viroids in this family, implying the possibility of similar functions. However, the presence of many distinct loops in different viroids evokes the question of whether there are viroid species-specific motifs for trafficking in the same or different host species. Furthermore, structural motifs for trafficking in the members of family Avsunviroidae remain a major knowledge gap. Rodio *et al*. [28] recently reported the first trafficking analysis of *Peach latent mosaic viroid* (PLMVd) in peach, showing invasion of the shoot apical meristem (SAM) of peach by PLMVd through in situ hybridization. This contrasts with the inability of PSTVd to enter SAM in *N. benthamiana* and tomato [13, 29], suggesting the existence of some fundamentally different mechanisms for the trafficking of viroids in the two families. Whether RNA silencing plays a role in the restriction of PSTVd to traffic into the SAM and in a broader context of viroid trafficking remains an intriguing question [30]. Further studies on the chloroplastic viroids to identify the RNA motifs and cellular factors involved in trafficking will significantly enhance our knowledge of how viroids have evolved various mechanisms to establish infection in plants.

The current progress in viroid trafficking is simply the beginning of a long journey in search of all answers regarding how a viroid traffics from cell to cell in a host plant to establish systemic infection. Many questions regarding RNA motifs and cellular factors remain to be answered: What and how many structural motifs in a viroid function independently or coordinately to traffic across each of the cellular boundaries in a plant to establish systemic infection? Can the same motifs mediate trafficking across more than one type of cellular boundaries? What trafficking motifs are common for a group of viroids and what motifs are unique to a viroid? What are the contributions of short helices in trafficking? What cellular factors are involved in trafficking across each cellular boundary? What are the relative contributions of cytosolic versus plasmodesmal factors? Do all cellular factors function in a cell-specific manner or do some factors function universally for trafficking across multiple or all of cellular boundaries? What cellular factors are common across host species and what factors are unique to a particular host? Certainly, these are not the only questions important to understand viroid trafficking. As research progresses to provide some answers, more questions will likely arise.

The importance of answering these questions goes beyond understanding the evolution of viroid-host interactions for the establishment of systemic infection. It will provide fundamental insights into the evolution of the molecular machinery for the trafficking of endogenous RNAs and infectious RNAs in plants. This is based on the simple premise that without encoding proteins, a viroid RNA most likely has evolved structural motifs to tap into the endogenous trafficking machinery to spread within an infected plant. Indeed, increasing evidence indicates that intercellular trafficking of RNAs plays important roles in physiological and developmental processes in plants [31–33]. Numerous mRNAs are detected in the phloem sap of several plant species [34–38]. Experimental studies showed that long-distance transport of some of these mRNAs can regulate development [39, 40]. A notable example is long-distance transport of potato *StBEL5* mRNA from shoot into stolon tips to regulate tuber formation [41]. The recent identification of the phloem-mobile pumpkin RBP50 ribonucleoprotein complex provides further compelling evidence in support of the broad significance of RNA trafficking in plant biology [27]. Infectious RNAs such as viroid and viral RNAs apparently utilize the endogenous trafficking system to spread within a plant to establish systemic infection [1, 2, 42–45]. A recent study showed that, in the absence of viral replication, some viral RNAs can traffic systemically, possibly by utilizing endogenous plant pathways [46]. Gene silencing signals, which likely have an RNA component, traffic intercellularly to trigger systemic silencing in plants and many other organisms as a means of gene regulation and antiviral defense [47, 48].

Intriguingly, numerous circulating nucleic acids including RNAs have been found in the human plasma and serum under healthy and diseased conditions, with their functions and mechanisms of entering and exiting the cells remaining to be understood [49]. Exosomes, membrane vesicles of endocytic origin that are released into extracellular space by many types of mammalian cells, contain mRNAs and microRNAs and can transfer these RNAs between different cells, potentially as a means of intercellular exchange of genetic information [50].

These observations suggest that cell-to-cell trafficking of RNAs underlies many biological processes. Elucidating the underlying mechanisms is crucial to understand gene regulation as well as host-pathogen interactions at the organismal level. Outstanding mechanistic questions for all RNA trafficking include: What RNA motifs direct trafficking between different cells? Do these motifs function individually or in combination to direct trafficking across a cellular boundary? What cellular factors are involved in the recognition and trafficking of an RNA? Viroids are highly tractable and simple models to investigate these questions [44]. The small genome size of a viroid RNA allows exhaustive mutagenesis to identify all of the RNA motifs for trafficking. The relatively simple and well-understood secondary structure of a viroid RNA permits detailed structural studies of trafficking motifs at the secondary and tertiary levels. Knowledge of all motifs may establish a library of trafficking motifs that allow searches of similar motifs in other RNAs. Viroid infection involves trafficking across all cellular boundaries, making it productive to identify motifs for each of cellular boundaries. All protein factors will be of cellular origin, unlike viruses where viral proteins also are involved making it necessary to separate viral and cellular proteins.

It is important to point out that we cannot rule out the possibility that viroid-host interactions may drive the evolution of some machinery specifically suited for viroid trafficking. For instance, a viroid RNA might interact with a cellular protein in such a way that the protein changes its normal cellular function or gains a new function to simply serve the purpose of spreading the viroid. On the other hand, an outstanding question is whether any plants have evolved mechanisms to actively restrict the intercellular trafficking of a viroid as a defense response, while allowing certain level of intracellular replication. These issues have profound significance in understanding the evolution of pathogen-host interactions.
